# Incomplete immunization coverage and its related factors among children aged 1-5 years: evidence from the Indonesia Family Life Survey 5

**DOI:** 10.4314/ahs.v26i2.6

**Published:** 2026-06

**Authors:** Erni Astutik, Tika Dwi Tama, Jauhari Oka Reuwpassa, Atik Choirul Hidajah, Muhammad Taaha Kashif, Rudi Safarudin

**Affiliations:** 1 Department of Epidemiology, Biostatistics, Population Studies, and Health Promotion, Faculty of Public Health, Universitas Airlangga, Surabaya, East Java, Indonesia; 2 Department of Public Health, Faculty of Sport Science, State University of Malang, Malang, East Java, Indonesia; 3 Dinoyo Primary Health Center, Malang District Health Office, Malang, East Java, Indonesia; 4 Research Group for Health and Wellbeing of Women and Children, Universitas Airlangga, Indonesia; 5 West Virginia School of Osteopathic Medicine, Lewisburg, West Virginia, USA; 6 Department of Pharmacy, Tadulako University, Palu, Central Sulawesi, Indonesia; 7 Prescription Drug Misuse Education and Research (PREMIER) Center, College of Pharmacy, University of Houston, Texas, USA

**Keywords:** Child health, vaccination, vaccine preventable disease, infectious disease prevention

## Abstract

**Background:**

The coverage of completeness of immunization is still an important problem in many countries, including Indonesia.

**Objectives:**

The aims of this study were to determine factors related to get incomplete immunization in Indonesia.

**Methods:**

The data were collected from the Indonesian Family Life Survey (IFLS) 5 with a sample of 782 women childbearing age (15-49 years) who had children aged 1–5 years old in Indonesia. The data was analyzed by using multiple logistic regressions.

**Results:**

Mother with lower education had 1.91 times greater odds for having incomplete immunization status compared with higher education (AOR=1.91, 95%CI 1.11-3.28). Mother who working contributed to improving incomplete immunizations compared with respondent who did not work (AOR=1.60, 95%CI 1.12-2.29). Mothers who lived in urban area had 1.48 higher odds to have incomplete immunization status in children compared to mother who lived in rural area (AOR=1.48, 95%CI 1.09-2.02). Husbands and both partners who have the power to make health decisions have lower odds of having a child whose immunizations are incomplete compared to only the mother who makes health decisions (AOR father =0.39, 95%CI=0.21-0.73; AOR both=0.62, 95%CI=0.42-0.92).

**Conclusion:**

Children's age, mothers' education and occupation, and health decision maker were related to incomplete immunization status.

## Introduction

Immunization is an empirically substantiated strategy for regulating infectious diseases, with projected efficacy in averting 3.5 to 5 million fatalities annually^1^. In juveniles, this prophylactic measure yields safeguarding effects against afflictions, which include pneumonia attributed to Haemophilus influenzae type B and Streptococcus pneumoniae, enteric disorders stemming from rotaviral origins, and infectious namely measles, poliomyelitis, diphtheria, tetanus, and pertussis^2^.

Insufficient vaccination rates due to poor immunization measures coupled with the emergence of diseases constitute disconcerting trajectories within developing nations. One such case is Indonesia, where the Ministry of Health inaugurated the Immunization Development Program in 1956, focusing on pediatric demographics, in a concerted endeavor to curtail the prevalence of diseases that could be mitigated through immunization. The targeted ailments encompassed tuberculosis, diphtheria, pertussis, measles, poliomyelitis, tetanus, and hepatitis B. Directive No. 42 of 2013 from the Minister of Health of the Republic of Indonesia described the program's scope, encompassing HB-0, Bacillus Calmette Guerin (BCG), Diphtheria Pertussis Tetanus-Hepatitis B (DPT-HB), or Diphtheria Pertussis Tetanus-Hepatitis B-Hemophilus Influenza type B (DPTHB-Hib), along with polio and measles^3^.

Indonesia's Basic Health Research of 2018 underscored a decline in complete immunization coverage from 59.2% in 2013 to 57.9% in 2018. About 33% received partial immunization, while 9.2% remained devoid of any immunization, citing various rationales such as aversion to vaccines, frequent bouts of illness, familial dissent, restricted access to immunization services, lack of guidance on immunization facilities, and time constraints^4^,^5^.

Numerous investigations have expanded on various factors influencing comprehensive immunization status among pediatric populations. Leveraging data from the Indonesia National Socioeconomic Survey spanning 2008, 2011, and 2013, a study determined noticeable trends. In the survey, households with maternally advanced age, educated mothers, and a healthcare professional exhibited a greater likelihood of full immunization status in children^6^. Expanding this investigation, an exploration of the determinants of immunization status among children under the age of 2 in Sumatra using Indonesia's 2020 National Socioeconomic Survey revealed additional determining factors. Notably, these include place of residence, mother's education, mother's employment status, and place of birth. These factors have a statistically significant relationship with the achievement of complete immunization status in children^7^.

Further insights were garnered from an autonomous investigation conducted via the Indonesia Demographic Health Survey. This inquiry underscored the interplay between economic status, utilization of antenatal care, the site of parturition, and their collective bearing on the children's immunization status^8^. The composite findings from these investigations suggest an interdependent relationship between women's pivotal role and children's health^9^.

Although preceding research has identified abundant variables, it is apparent that a predominant emphasis has been placed on maternal determinants. Consequently, this study aims to analyze the association between incomplete immunization status among children aged 1-5 years in Indonesia and an array of multifaceted elements: maternal and paternal factors, residence, household condition, religiosity, decision-maker, and subjective well-being. The overarching objective is to relationships underpinning incomplete immunization status within this specific demographic cohort.

## Methods

### Study design

This study was a cross-sectional survey among a representative sample of children aged 1–5 years from households in both urban and rural localities in Indonesia. The data were collected from the fifth wave of the Indonesian Family Life Survey (IFLS-5). The IFLS-5 was fielded between late October 2014 and the end of April 2015.

### Population Sampling

The Indonesia Family Life Survey (IFLS) constitutes a longitudinal inquiry, inaugurated with its inaugural wave during 1993-1994. The survey's scope encompasses a representative sample, encapsulating approximately 83% of Indonesia's population living across 13 of 26 provinces in the nation^10^.

For the IFLS5, the sampling framework emanated from the cohorts of IFLS1, IFLS2, IFLS2+, IFLS3, and IFLS4, thereby fostering a continuum of longitudinal data. In the case of IFLS1, the sampling procedure adhered to a stratification model, delineating provinces and demarcating urban-rural locales, then random samples were taken within these strata. The execution of IFLS 5 transpired through recontacting prior participants. The contact rate back at IFLS 5 among IFLS 1 was 76% (including mortality).

The demographic under scrutiny within this investigation comprises women within the childbearing age bracket of 15 to 49 years, all of whom have offspring ranging from one to five years in age at the juncture of the interview. The eligible population of this study was 782 participants after excluding the missing data. All of them were study participants.

### Data collection

Immunization status refers to participants who have immunized children. The immunization development program includes one BCG immunization, DPT-HB immunizations, polio immunizations, and measles immunization. Individuals who had not administered the full spectrum of immunization development programs to their children were categorized as possessing an “incomplete immunization status”.

Our study encompassed an array of parameters for comprehensive evaluation. These included age children (months), children's sex (male/female), and health status of children (very healthy, somewhat healthy, somewhat unhealthy, very unhealthy). In addition, we measured maternal factors including mother's age (years), mother's education (lower, medium, higher), mother's occupation (working and not working), ethnicity (Javanese and non-Javanese), residence (rural and urban), household size (household number <4 or ≥ 4), number of children, religiosity (very religious, somewhat religious, rather religious, not religious), health decision maker (mother, husband, both, and others), subjective wellbeing (satisfied, somewhat satisfied, not satisfied), place of birth (health facility, non-health facility).

Additionally, we measured father's age (years), father's education (lower, medium, higher), and father's occupation (working and not working).

### Data Analysis

Descriptive statistics were used to describe the features of the data. The effect of potential predictors on immunization was analyzed using multivariable logistic regression. Variables that had p value < 0.25 in bivariate analysis were entered into the multivariate model. Variables were identified as having an association with immunization status if the p value < 0.05 (95% confidence interval). All the data were analyzed using STATA 14.

## Results

In this study, 47.19% of the participants reported complete immunization status of their children. The average age (SD) of the children under examination stood at 30.80 (13.53) months). Slightly more than half of the children were male (51.28%), and a majority of participants reported their children's health status as somewhat unhealthy (60.23%).

Regarding maternal attributes, the average age of mothers (SD) was 27.81 (5.23) years. Predominantly, mothers possessed medium levels of education (68.80%) and were non-working (76.60%). A significant proportion resided in urban localities (63.17%) and identified as Javanese ethnicity (52.69%). Notably, a substantial portion belonged to larger households comprising more than four members (67.77%). The average number (SD) of children per mother was 1.49 (0.66). Furthermore, a mere 2.17% of mothers reported not religious. A notable finding was that over half of the health decisions were jointly made by both parents (mother and father), with only 7.80% expressing dissatisfaction with their lives. Additionally, a mere 17.52% of mothers indicated giving birth outside of health facilities (Table 1). Conversely, the average age (SD) of fathers stood at 31.83 (5.58) years. Analogous to mothers, the majority of fathers possessed medium levels of education (68.80%), and a substantial proportion were employed (94.25%). (See Table 1 for further details.)

**Table 1:** Distribution of variables (N=782)

Variables	n	%	Mean (SD)
Immunization Status			
Completed	369	47.19	
Incomplete	413	52.81	
Children's age (months)			30.80 (13.53)
Children's sex			
Female	381	48.72	
Male	401	51.28	
Health status of children			
Somewhat Healthy	187	23.91	
Somewhat Unhealthy	471	60.23	
Very Unhealthy	124	15.86	
Mother's age(years)			27.81 (5.23)
Mother's education			
Lower	124	15.86	
Medium	538	68.80	
Higher	120	15.35	
Mother's Occupation			
Working	183	23.40	
Not working	599	76.60	
Ethnicity			
Javanese	412	52.69	
Non-Javanese	370	47.31	
Residence			
Rural	288	36.83	
Urban	494	63.17	
Household number			
<4	252	32.23	
≥4	530	67.77	
Number of children			1.49 (0.66)
Religiosity			
Very religious	85	10.87	
Somewhat religious	503	64.32	
Rather religious	177	22.63	
Not religious	17	2.17	
Health decision-maker			
Mother	145	18.54	
Husband	62	7.93	
Both	563	71.99	
Others	12	1.53	
Subjective wellbeing			
Satisfied	378	48.34	
Somewhat Satisfied	343	43.86	
Not satisfied	61	7.80	
Place of birth			
Health facility	645	82.48	
Non health facility	137	17.52	
Father's age (years)			31.83 (5.58)
Father's education			
Lower	135	17.26	
Medium	523	66.88	
Higher	124	15.86	
Father's occupation			
Working	737	94.25	
Not working	45	5.75	

Based on Table 2, the mother's age, mother's occupation, residential area, number of children, and father's age were significantly associated with incomplete immunization status in children aged 1-5 years.

**Table 2:** Distribution of independent and outcome variables (N=782)

Variables	Immunization Status				p-value
	Incomplete		Completed		
	n	%	n	%	
Children's age (months)					0.146
Children's sex					
Female	204	53.34	177	46.46	0.690
Male	209	52.12	192	47.88	
Health status of children					
Somewhat Healthy	105	56.15	82	43.85	0.540
Somewhat Unhealthy	242	51.38	229	48.62	
Very Unhealthy	66	53.23	58	46.77	
Mother's age(years)					0.001*
Mother's education					
Lower	74	59.68	50	40.32	0.248
Medium	277	51.49	261	48.51	
Higher	62	51.67	58	48.33	
Mother's Occupation					
Working	111	60.66	72	39.34	0.015*
Not working	302	50.42	297	49.58	
Ethnicity					
Javanese	211	51.21	201	48.79	0.344
Non-Javanese	202	54.59	168	45.41	
Residence					
Rural	137	47.57	151	52.43	0.025*
Urban	276	55.87	218	44.13	
Household number					
<4	127	50.40	125	49.60	0.351
≥4	286	53.96	244	46.04	
Number of children					0.005*
Religiosity					
Very religious	45	52.94	40	47.06	0.797
Somewhat religious	265	52.68	238	47.32	
Rather religious	92	51.98	85	48.02	
Not religious	11	64.71	6	35.29	
Health decision-maker					
Mother	89	61.38	56	38.62	0.059
Husband	26	41.94	36	58.06	
Both	292	51.87	271	48.13	
Others	6	50	6	50	
Subjective wellbeing					
Satisfied	199	52.65	179	47.35	0.176
Somewhat Satisfied	175	51.02	168	48.98	
Not satisfied	39	63.93	22	36.07	
Place of birth					
Health facility	342	52.78	306	47.22	0.965
Non health facility	71	52.99	63	47.01	
Father's age (years)					0.021*
Father's education					
Lower	70	51.85	65	48.15	0.784
Medium	274	52.39	249	47.61	
Higher	69	55.85	55	44.35	
Father's occupation					
Working	386	52.37	351	47.63	0.320
Not working	27	60	18	40	

* The significance level used is 5% (p < 0.05)

After being controlled by covariate variables, children's age, mothers' education, mothers' occupation, residence, and health decision-maker were all significantly associated with incomplete immunization status. Every increase in the children's age (months) would reduce the risk of getting incomplete immunization. Mothers with lower education had 1.91 times greater odds of having incomplete immunization status for their children than mothers with higher education (AOR=1.91, 95%CI 1.11-3.28, p=0.019). Working mother was associated with 1.60 times increase in the odds of incomplete immunization status for children aged 1-5 years, compared to respondents who did not work (AOR=1.60, 95%CI 1.12-2.29, p=0.019). Mothers residing in urban areas exhibited 1.48 times higher odds of incomplete immunization status in their children than those in rural areas (AOR=1.48, 95%CI 1.09-2.02, p=0.012). Parents, whether fathers only or both parents together, making health decisions were associated with reduced odds of having a child with incomplete immunization, in contrast to mothers making health decisions individually (AOR father=0.39, 95%CI=0.21-0.73, p=0.003; AOR both=0.62, 95%CI=0.42-0.92, p=0.016).

## Discussion

### General Finding

This paper investigated the determinants of children's immunization status in Indonesia aged 1-5 years using IFLS 5 data. The overarching analysis revealed a nuanced understanding that emerged from the multivariable analysis. Overall, the percentage of children who got complete immunizations was 47.19%. Based on multivariable analysis, however, every increase in the children's age (months) would reduce the risk of getting incomplete immunization. Mothers with lower education had greater odds of having incomplete immunization status in their children than mothers with higher education. Mothers who worked had a lower likelihood of incomplete immunizations in their children aged 1-5 years than mothers who did not work. Mothers who lived in urban areas had higher odds of having incomplete immunization status in their children than mothers who lived in rural areas. Families in which both parents make health decisions and families in which only the husband makes health decisions both had lower odds of having incomplete immunizations in their children than families in which only the mother makes health decisions.

### Comparison with Other Studies and Possible Mechanisms

#### Children's Age

The results demonstrated that an increase in children's age decreased complete immunization likelihood. In this study, if the date of each immunization was not recorded on the vaccination card, the immunization status was evaluated based on parental recall, especially the mothers^11^. Previous studies suggested that most parents could report immunization status correctly for children under six months compared to older children^12^-^14^. The mother's accuracy in remembering her child's immunization status decreases as the child ages^15^. The average age of the children in this study was 30 months. Parents or mothers might forget about the vaccines that their children received. They also had difficulties recalling the number of doses that their children had received. The error that occurred due to maternal recall was greater when the mother was asked to report the vaccine status with more than two doses^14^,^15^. Most mothers tend to underestimate reporting DPT and Polio vaccine doses^15^. Because of the underreported vaccination doses, the children's immunization status was classified as incomplete.

Furthermore, the additional children's age had a higher possibility of incomplete immunization due to the increased retention of vaccination cards. The higher the vaccination card retention, the less accurate immunization data can be collected. Studies conducted in Pakistan and Cameroon showed that older children had lower odds of keeping vaccination cards^16^,^17^. The odds of vaccination card retention by mothers or caregivers ranged from 46% to 90% if their child was over two years old compared to those whose child is under two years of age^16^,^17^. In Indonesia, immunizations are recorded on vaccination cards which are for families to keep. Despite this, parents may not prioritize proper safekeeping of the vaccination card even though their children have received complete immunization^17^.

#### Mother's education

Results of this study indicate low education level mothers have an increased tendency to discourage their children from receiving complete immunization compared to higher education level mothers. The findings of this research are consistent with other studies stating that higher education is associated with a greater likelihood of having children who are fully immunized^18^-^22^. Lack of knowledge about immunization can lead to misconceptions and false beliefs about vaccination^18^,^23^,^24^. Some beliefs that influence immunization include religious permissibility of vaccines^25^-^27^ and sickness resulting from vaccines^19^,^25^.

Moreover, educated mothers are more likely to engage in Antenatal Care (ANC)^26^, which may increase education regarding vaccines among healthcare providers. This study also found that mothers with medium education had higher odds to get incomplete immunization status in children compare to mothers with higher education but the relationship was not significant. It can be explained that mother with medium and higher education generally had good literacy. Therefore, they can access information well.

#### Mother's employment status

The results of this study highlight that incomplete immunization status is increased in families with working mothers compared to families without working mothers. These findings are consistent with other research, which states that working mothers have a lower likelihood of their children receiving complete immunization^27^-^29^. Other studies, however, have found that mothers working part-time are more likely to have children who receive complete immunization than mothers who work full-time^30^. One suggested explanation is that immunization schedules often coincide with mothers' working hours^33^, resulting in working mothers having limited opportunities to provide immunization to their children^31^. Furthermore, mothers who do not work are more likely to focus on their children, enabling them to seek immunization for their children^7^,^31^,^32^.

#### Residential Status

Mothers residing in urban areas had higher odds of having incomplete immunization status in children than in rural areas. This study aligns with other research that states that children living in rural areas are more likely to have complete immunization status than those living in urban areas^7^. This result conflicts with other studies, however, as one study indicated children living in rural areas are more prone to having incomplete immunization status^33^. Other research findings have suggested that geographic location is not associated with immunization status^34^. The dissimilarities across research outcomes may be ascribed to an array of factors, including cultural disparities, religious convictions, the accessibility of healthcare resources, and the availability of medical facilities^35^,^36^. These diverse elements can fluctuate significantly across distinct geographic settings and contribute to the fluctuations in immunization status exhibited among various population groups. The intricate interplay of these multifaceted factors underscores the complexity inherent in comprehending and addressing immunization status disparities.

#### Decision-making power in household

The likelihood of complete immunization was lower among children who had their father or both their parents as the health-related decision-maker in the household than among children whose mother was the decision-maker. A study conducted in multiple countries in South Asia and Ethiopia found a similar finding^37^-^40^. A mother who does not have the autonomy to be the decision-maker in the household tends to have incompletely immunized children^37^-^40^. One study attributed this finding to lower awareness of the father for his child's health compared to the mother^40^. Our study also found that other people as the health-related decision maker in household was lower odds to get complete immunization status in children compared to mother who made the decision maker. This finding showed that mother had a significant role in ensuring their children's essential health needs was received, including complete immunization. Having a joint decision with the husband regarding the child's health also had higher odds of having incomplete immunization status in the child. Even though the decision may be made together, the mother opted to wait for the father's approval or permission due to his status as head of the household^39^.

## Strengths and Limitations

This study has strengths in its methodology owing to its substantial sample size, which enabled rigorous testing of numerous associations with robust statistical power. The utilization of secondary data derived from the IFLS is a notable strength, encompassing a representative spectrum of 83% of Indonesia's population, thereby conferring unbiased insights into population-wide estimates. An additional advantage rests in utilizing the most current national survey data available, which augments the study's contemporaneity and relevance.

However, we acknowledged certain limitations inherent in the study design. The cross-sectional nature of the investigation engenders a susceptibility to temporal ambiguity, potentially obscuring the establishment of causal relationships. Moreover, the study's focus on infants aged 1-5 years introduces the potential for information bias. Despite these limitations, the study contributes valuable insights into immunization status determinants and underscores the need for cautious interpretation within the context of its design constraints. Additionally, we used the latest survey of IFLS conducted in 2014 and 2015 and did not consider family income in this study.

## Policy Implications

The significance of addressing the proportion of children who have not achieved full immunization cannot be understated. The immunization program and stakeholders should also consider monitoring children who are not fully immunized or have not received any immunizations. National immunization programs need to be improved. They especially need to be promoted to parents unwilling to give their children immunizations or incomplete immunizations. In addition, it is necessary to identify mothers whose children at risk do not complete the immunization schedule and educate them about the importance of complete vaccination. The government or companies should be able to provide policies for mothers with babies to immunize their children. Besides, we need to do further research to measure other variables related to immunization status.

## Conclusion

In this study, mothers' education level and occupation were significantly associated with complete immunization status in children 1-5 years old. Immunization promotion programs should target more working mothers and those with low education level. In addition, immunization-related health promotion and educational programs are needed to be improved in rural areas.

## Figures and Tables

**Table 3:** Unadjusted and Adjusted odds ratios of determinant of incomplete immunization status in children aged 1–5 years in Indonesia

Variables	Univariate				Multivariate			
	OR	95% CI		p value	AOR		95% CI	p value
		Lower	Upper		Lower		Upper	
Children's age (months)	0.99	0.98	1.003	0.146	0.989	0.978	0.999	0.039*
Children's sex								
Female	Ref							
Male	0.94	0.71	1.25	0.690				
Health status of children								
Somewhat Healthy	Ref							
Somewhat Unhealthy	0.83	0.59	1.16	0.269				
Very Unhealthy	0.89	0.56	1.40	0.612				
Mother's age(years)	1.05	1.02	1.08	0.001*				
Mother's education								
Lower	1.38	0.83	2.30	0.208	1.91	1.11	3.28	0.019*
Medium	0.99	0.67	1.48	0.972	1.31	0.86	2.00	0.209
Higher	Ref				Ref			
Mother's Occupation								
Working	1.52	1.08	2.12	0.016*	1.60	1.12	2.29	0.019*
Not working	Ref				Ref			
Ethnicity								
Javanese	Ref							
Non-Javanese	1.15	0.86	1.52	0.344				
Residence								
Rural	Ref				Ref			
Urban	1.40	1.04	1.87	0.025*	1.48	1.09	2.02	0.012*
Household number								
<4	Ref							
≥4	1.15	0.85	1.56	0.351				
Number of children	1.37	1.10	1.70	0.005*				
Religiosity								
Very religious	Ref							
Somewhat religious	0.99	0.62	1.57	0.965				
Rather religious	0.96	0.57	1.62	0.884				
Not religious	1.63	0.55	4.81	0.376				
Health decision-maker								
Mother	Ref				Ref			
Husband	0.45	0.25	0.83	0.011*	0.39	0.21	0.73	0.003*
Both	0.68	0.47	0.98	0.041*	0.62	0.42	0.92	0.016*
Others	0.63	0.19	2.05	0.442	0.60	0.18	2.01	0.404
Subjective wellbeing								
Satisfied	Ref							
Somewhat Satisfied	0.94	0.70	1.26	0.663				
Not satisfied		1.59	0.91	2.79	0.103			
Place of birth								
Health facility		Ref						
Non health facility		1.03	0.71	1.49	0.885			
Father's age (years)		1.03	1.004	1.06	0.022			
Father's education								
Lower		0.86	0.53	1.40	0.541			
Medium		0.88	0.59	1.30	0.514			
Higher		Ref						
Father's occupation								
Working		0.73	0.40	1.35	0.321			
Not working		Ref						

## Data Availability

Data could be accessed on https://www.rand.org/labor/FLS/IFLS.html
